# Primary Gingival Squamous Cell Carcinoma With Secondary Extension Into the Maxillary Sinus, Orbit, and Skull Base: A Case Report

**DOI:** 10.7759/cureus.107016

**Published:** 2026-04-14

**Authors:** Alireza Izadian Bidgoli, Amanda Pina, Shabnam Yazdanpanah, Gabriel A Saavedra, Guillermo Rame, Damian Casadesus

**Affiliations:** 1 Internal Medicine, American University of the Caribbean School of Medicine, Cupecoy, SXM; 2 Internal Medicine, Jackson Memorial Hospital, Miami, USA; 3 Internal Medicine, Ross University School of Medicine, Two Mile Hill, BRB

**Keywords:** delayed diagnosis, gingival squamous cell carcinoma, perineural invasion, skull base extension, social determinants of health

## Abstract

Gingival squamous cell carcinoma (GSCC) is an uncommon oral malignancy that may mimic benign periodontal disease, often leading to delayed diagnosis and advanced stage presentation. We present a 68-year-old man with primary GSCC demonstrating extensive secondary invasion into the maxillary sinus, orbit, and skull base. He presented with progressive weakness, poor oral intake, cachexia, right facial numbness, and visual disturbances. His history was notable for a prolonged absence of medical and dental care due to financial barriers. Imaging revealed a large, destructive mass with orbital apex, cavernous sinus, and skull base involvement, consistent with aggressive perineural and intracranial spread, with regional metastasis to a level II cervical lymph node and distant osseous metastasis to the occipital bone. Given the extent of disease, the tumor was deemed unresectable and managed with systemic therapy, radiation, and corticosteroids for compressive optic neuropathy. This case highlights the aggressive potential of GSCC when diagnosis is delayed and emphasizes the critical role of early recognition and access to preventive care in improving outcomes.

## Introduction

Gingival squamous cell carcinoma (GSCC) is a relatively uncommon malignancy of the oral cavity, accounting for less than 10% of oral cancers, and often presents a diagnostic challenge due to its clinical resemblance to benign periodontal disease [1]. Early-stage GSCC frequently mimics common dental conditions, including localized periodontitis, gingival inflammation, and denture-related trauma, presenting with nonspecific findings such as gingival erythema, ulceration, tooth mobility, and alveolar bone loss [1,2]. As a result, delays in diagnosis are common, with studies reporting that a substantial proportion of gingival carcinomas are present for extended periods before biopsy is performed, and many cases are initially misdiagnosed [3,4]. Delays in diagnosis are common, with studies reporting that gingival carcinomas may remain unrecognized for several months, and in some cases over six months, before biopsy is performed [3,4].

Delayed recognition of GSCC has significant clinical consequences. The tumor demonstrates a strong propensity for early invasion into adjacent osseous structures, particularly the alveolar bone and maxilla, with a high percentage of cases showing bone involvement at the time of diagnosis [2,5]. With continued progression, untreated lesions may extend beyond the oral cavity into the maxillary sinus and surrounding craniofacial compartments, including the orbit and skull base, where involvement of critical neurovascular structures can render the disease unresectable and substantially worsen prognosis.

Social determinants of health play a critical role in the timing of diagnosis and outcomes in oral cavity malignancies. Limited access to healthcare, lack of insurance, and reduced utilization of preventive dental services are strongly associated with advanced-stage presentation [6]. Access to routine dental care is particularly important for early detection, as patients who rarely or never undergo dental evaluation have significantly increased odds of presenting with advanced disease, while regular dental visits are associated with earlier-stage diagnosis and improved outcomes [7,8].

In this report, we present a case of primary GSCC with extensive secondary invasion into the maxillary sinus, orbit, and skull base, illustrating the catastrophic consequences of delayed diagnosis. This case emphasizes the importance of early recognition of subtle oral and craniofacial symptoms, the need to distinguish primary tumor origin from secondary extension in advanced disease, and the broader impact of healthcare access on oncologic outcomes.

## Case presentation

A 68-year-old man with no past medical history presented to the emergency department with progressive generalized weakness, poor oral intake, nausea, vomiting for one week, and a 14 kg weight loss from a baseline of 70 kg. He also reported a new onset of right facial numbness and orbital symptoms. The patient was unable to clearly specify the duration of the gingival mass; however, he reported a progressively enlarging lesion prior to presentation. He also reported a recent onset of orbital symptoms, including proptosis, developing over approximately one week. The patient reported that his last medical evaluation was five years prior and his last dental visit was three years prior.

On examination, he appeared chronically ill but in no acute distress. The HEENT (head, eyes, ear, nose, throat) exam demonstrated edema and a palpable mass in the right maxillary region with right-sided proptosis. Neurological exam demonstrated decreased sensation over the superior nasal and supraorbital distribution, localizing to involvement of the ophthalmic division of the trigeminal nerve, CN V1. Cardiopulmonary exam was unremarkable aside from sinus bradycardia. Ophthalmologic evaluation revealed right-eye proptosis, conjunctival injection with inferior chemosis, diffuse extraocular movement restriction, decreased visual acuity (20/200 OD improving to 20/50). The left eye had chronic vision loss from remote trauma. Fundoscopic examination showed a normal optic disc without edema.

**Figure 1:**
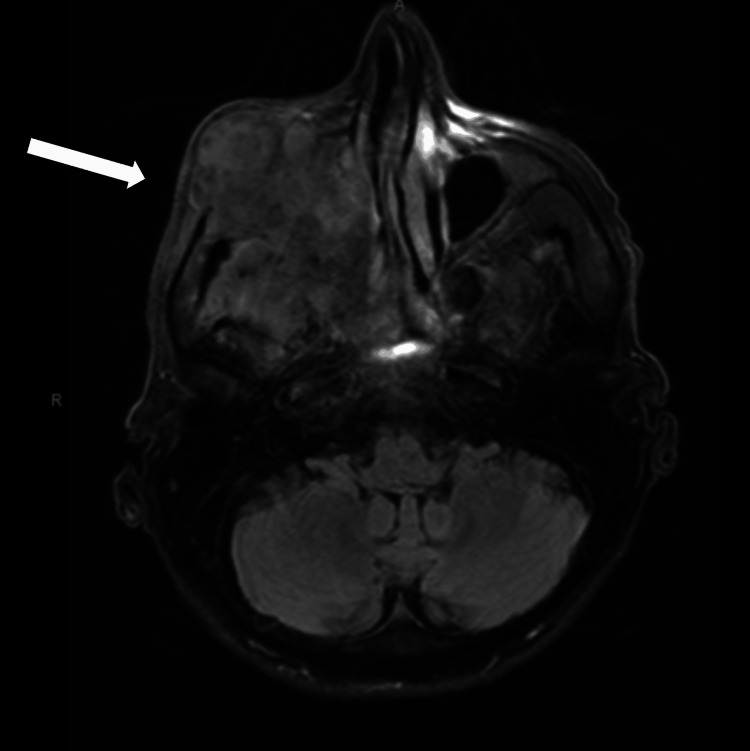
MRI of the brain with and without contrast. MRI brain revealed a large 7.1x5.7x5.2 cm mass centered in the right maxillary sinus with invasion of the nasal cavity, ethmoid and sphenoid sinuses, orbital apex, cavernous sinus, masticator space, multiple skull base foramen, and intracranial extension.

MRI of the brain with and without contrast demonstrated a large, heterogeneously enhancing necrotic mass centered in the right maxillary sinus measuring 7.1x5.7x5.2 cm (Figure 1). The mass showed destruction of the maxillary sinus walls with extension into the nasal cavity, ethmoid and sphenoid sinuses, and right orbit, including invasion of the extraocular muscles and involvement of the orbital apex and optic nerve. There was extension into the cavernous sinus with encasement of the internal carotid artery, as well as perineural spread along the trigeminal nerve into Meckel’s cave and the pterygopalatine fossa. Additional involvement included skull base foramina (foramen rotundum and ovale), hard palate, soft palate, nasopharynx, and masticator space. No acute intracranial hemorrhage or hydrocephalus was identified. Imaging also demonstrated metastasis to the right occipital bone and a 2.6 cm level II cervical lymph node.

**Figure 2:**
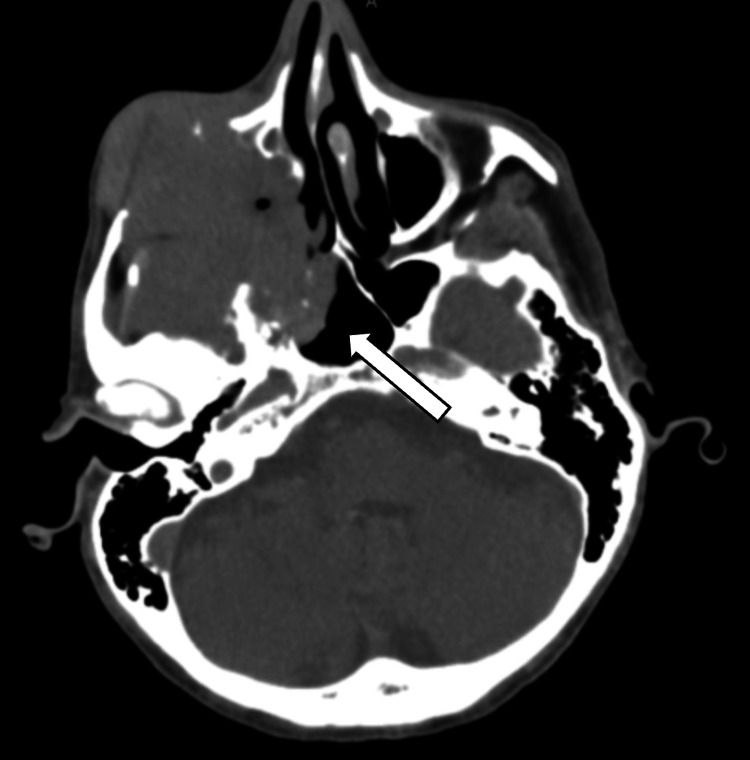
CT Brain revealing a large infiltrative mass on the right maxillary sinus. Mass centered in the right maxillary/maxillary sinus with mass effect, osseous erosion, and vascular encasement as described above. The mass invades the right anterior middle cranial fossa abutting the right inferior temporal lobe, right sphenoid sinus, and right orbit. The mass demonstrates erosion of the right maxilla, maxillary sinus walls, and multiple other osseous structures.

A CT scan of the head revealed large multicomponent multilobulated enhancing destructive/infiltrative mass centered in the right maxillary/maxillary sinus with mass effect, osseous erosion, and vascular encasement as described above (Figure 2). Further CT scan of the abdomen and pelvis demonstrated lytic lesions on the acetabulum suspicious for metastatic lesions.

Biopsy of the right upper gingival lesion confirmed invasive moderately differentiated keratinizing SCC with stromal invasion, consistent with a primary gingival origin. The extent of disease involving the maxillary sinus and adjacent structures reflects secondary tumor extension.

Electrocardiogram (EKG) revealed sinus bradycardia with occasional premature ventricular complexes (PVCs) and low-voltage QRS complexes. Laboratory studies showed mild anemia and hypoalbuminemia consistent with cancer-related cachexia.

Multidisciplinary tumor board discussion

The case was reviewed by a multidisciplinary tumor board including otolaryngology, neurosurgery, medical oncology, radiation oncology, and ophthalmology. Given extensive skull base, cavernous sinus, and orbital involvement, the tumor was deemed unresectable due to infiltration of critical neurovascular structures and intracranial extension.

The consensus recommendation was definitive chemoradiation. High-dose corticosteroids were initiated due to concern for compressive optic neuropathy, and outpatient systemic therapy planning was coordinated.

The patient was started on high-dose oral prednisone with taper for compressive optic neuropathy and planned for outpatient chemoradiation. During hospitalization, symptoms of weakness and dizziness were monitored; orthostatic vitals were evaluated. Nutritional supplementation was initiated for failure to thrive and cancer cachexia.

At discharge, he remained neurologically stable without acute intracranial complications. Vision in the right eye showed mild improvement in best corrected acuity, and the optic disc remained without edema. He was discharged with outpatient follow-up for initiation of definitive chemoradiation and further oncologic management.

## Discussion

The earliest indexed data for GSCC was acquired through a large retrospective series study with cases from 1967 through 1994 [9]. The study identified 300 gingival/alveolar ridge SCCs among 1,193 total oral SCCs [9].

According to a recent study of 419 cases, there was a rising incidence of GSCC between 2010 and 2020 with the posterior mandibular gingiva being the most affected site [4]. According to the study, the average age for diagnosis was 69.8 years with 62% of the patients having a previous history of smoking [4]. GSCC usually presents as an exophytic mass, but it can also present as an ulcerative, erythematous, or papillary lesion [3]. It can be a diagnostic challenge because it frequently mimics periodontal diseases [3].

Most GSCCs are moderately differentiated conventional keratinizing SCC with immunohistochemistry showing heterogeneous expression of CK1/10 and CK19 [3,4]. It was also found that vertical (upward) tumor spread from the alveolar ridge to the maxillary sinus floor, nasal fossa, and orbital floor were all independent prognostic factors that contribute to a poor outcome while horizontal (antero-posterior) extent along the alveolar ridge doesn’t affect the prognosis for the patients; radiologically, this helps suggest that prognosis could be poor if the tumor spreads to the maxillary sinus, nasal fossa, or orbital floor [10].

Comparative analysis of our case with the existing literature****

SCC of the oral cavity demonstrates significant heterogeneity in both clinical presentation and biological behavior. Large epidemiologic and clinicopathologic studies indicate that GSCC most commonly presents as a localized ulcerated lesion involving the mandibular or maxillary gingiva, frequently mimicking inflammatory periodontal disease or odontogenic pathology [2,3]. This diagnostic overlap can delay recognition of malignancy, particularly in early stages when symptoms are subtle. In contrast to the relatively localized presentations reported in many case series, the present case exhibited extensive craniofacial extension with orbital and skull base involvement at the time of evaluation, reflecting a markedly advanced disease pattern.

Aggressive local progression has been described in a limited number of reports involving rapidly enlarging head and neck SCC. Rapid tumor expansion with extensive destructive invasion illustrates the capacity of certain tumors to exhibit highly aggressive biological behavior and accelerated growth [11]. These observations underscore the biological variability of SCC and demonstrate that a subset of tumors may follow a particularly aggressive clinical course. In advanced cases, gingival SCC with extension into the maxillary sinus and adjacent sinonasal structures may spread along established anatomic pathways, including perineural routes and skull base foramina [12,13]. The extensive regional invasion observed in this case reflects these recognized patterns of tumor spread and highlights the complex anatomic relationships that often complicate treatment planning.

Radiologic assessment plays a critical role in defining tumor extent and determining resectability. In advanced cases with sinonasal extension, imaging frequently demonstrates destructive bone involvement, invasion of adjacent sinus cavities, and extension into surrounding soft tissue compartments [12]. Recognition of perineural tumor spread is particularly important, as involvement of cranial nerve pathways is associated with more aggressive disease and poorer outcomes. These imaging features are essential for accurate staging and often guide the decision between surgical and nonsurgical management. Perineural spread in SCC occurs through tumor cell migration along the perineural space, facilitated by complex interactions between tumor cells and neural elements, including neurotrophic factors, adhesion molecules, and extracellular matrix remodeling, which promote directional tumor growth along nerve sheaths [13-15].

Histopathologically, most oral cavity SCCs demonstrate keratinizing malignant squamous epithelium with varying degrees of differentiation. Moderately differentiated tumors, as identified in this case, represent a commonly reported subtype and are generally associated with intermediate tumor behavior [16]. Increasing attention has also been directed toward the molecular mechanisms underlying SCC progression. Studies have identified recurrent genetic alterations involving tumor suppressor pathways, particularly mutations in TP53 and deletions of CDKN2A, which contribute to malignant transformation and aggressive tumor growth [17]. These alterations have also been associated with more aggressive tumor behavior, higher rates of local invasion, and poorer clinical outcomes in head and neck SCC, highlighting their potential prognostic significance [18]. Although molecular testing is not routinely required for diagnosis, these findings continue to inform emerging research on targeted therapeutic strategies.

Therapeutic decision-making in head and neck SCC is largely determined by disease stage, anatomic involvement, and the feasibility of achieving complete surgical resection. According to established treatment guidelines, surgical resection remains the primary treatment modality for localized disease, often followed by adjuvant radiation therapy depending on risk factors [19]. However, when tumors demonstrate extensive invasion of critical neurovascular structures, as in this case, definitive chemoradiation becomes the preferred therapeutic approach. Similar management strategies have been described in advanced cases with the involvement of skull base, where surgical intervention is not feasible [20].

Delayed diagnosis and barriers to healthcare access are key contributors to advanced-stage presentation in oral and head and neck cancers. Population-based studies have demonstrated that individuals with limited access to routine dental care or preventive medical services are more likely to present with later-stage disease [6]. Regular dental examinations are associated with earlier detection of oral malignancies and improved staging at diagnosis [7]. This emphasizes the critical role of preventive care and early recognition in improving oncologic outcomes.

Prognosis in GSCC varies significantly by stage at presentation. Early-stage, localized disease is generally associated with favorable outcomes, with reported five-year survival rates often exceeding 70%-80% when treated with surgical resection with or without adjuvant therapy [10,19]. In contrast, locoregionally advanced disease, particularly with perineural invasion or regional lymph node involvement, is associated with substantially worse outcomes due to increased risk of local recurrence and spread along cranial nerve pathways [19]. The presence of perineural spread has been identified as an adverse prognostic factor and is frequently associated with more aggressive tumor behavior and decreased disease-free survival [10]. In cases of distant metastatic disease, prognosis is poor, with five-year survival rates markedly reduced and treatment largely focused on disease control and palliation [19]. These distinctions underscore the critical importance of early detection and intervention in improving survival outcomes [10,19].

What have we learned from this case

This case reframes advanced gingival SCC not simply as a consequence of delayed diagnosis, but as a time-sensitive neuro-oncologic process in which early cranial neuropathies and orbital symptoms represent critical inflection points in disease progression. In this patient, facial numbness, proptosis, and visual disturbances were not late incidental findings, but rather clinical markers of active perineural and skull base invasion, features that signal a transition from potentially curable disease to unresectable pathology.

A key clinical implication is that new-onset cranial nerve deficits in the setting of oral or maxillofacial complaints should be treated as oncologic red flags requiring immediate advanced imaging, even when initial symptoms may resemble benign dental or periodontal conditions. Failure to recognize this transition window may result in rapid progression along established anatomic pathways, including the trigeminal nerve, cavernous sinus, and skull base foramina, where surgical options become limited.

Equally important, this case highlights how structural barriers to healthcare access directly translate into biologic disease advancement. The prolonged absence of dental and medical care in this patient was not merely a social detail, but a determinant of tumor stage at presentation, reinforcing the role of preventive care as a modifiable factor in oncologic outcomes.

Finally, this case underscores the importance of early multidisciplinary engagement. Once critical neurovascular structures are involved, management shifts from surgical cure to disease control, with chemoradiation and adjunctive therapies serving primarily to preserve function and limit progression. Recognizing the narrow window before this transition is essential, as it represents the most meaningful opportunity to alter prognosis.

## Conclusions

This case illustrates the severe consequences of delayed recognition of GSCC, in which a potentially localized malignancy progressed to extensive invasion of the maxillary sinus, orbit, and skull base with metastatic spread, ultimately rendering the disease unresectable. It highlights the aggressive potential of GSCC, particularly when early symptoms are overlooked due to its resemblance to benign periodontal disease. The case also underscores the critical influence of social determinants of health, as limited access to medical and dental care contributed to advanced-stage presentation. Early recognition of atypical oral and craniofacial symptoms, prompt diagnostic evaluation, and improved access to preventive care remain essential to improving outcomes. Accurate distinction between primary tumor origin and secondary extension is equally important for appropriate staging and management in advanced head and neck malignancies. Early biopsy of persistent or atypical gingival lesions and prompt evaluation of new cranial neuropathies or orbital symptoms are critical to facilitate timely diagnosis and potentially improve outcomes.
